# Effects of Selective Sphingosine‐1‐Phosphate Receptor 1 Agonist, TRV045, on Evoked Pain Tests: An Exploratory, Four‐Way Cross‐Over Study in Healthy Volunteers

**DOI:** 10.1002/ejp.70314

**Published:** 2026-06-17

**Authors:** Wouter A. Bakker, P. Eijsvogel, Erica S. Klaassen, Jessica Kim, Ruihua Chen, Mark A. Demitrack, Albert Dahan, Marieke Niesters, Hemme J. Hijma, Geert Jan Groeneveld

**Affiliations:** ^1^ Centre for Human Drug Research Leiden the Netherlands; ^2^ Leiden University Medical Centre Leiden the Netherlands; ^3^ Trevena Inc. Chesterbrook Pennsylvania USA; ^4^ Department of Anesthesiology and Pain Medicine Erasmus Medical Center Rotterdam the Netherlands

**Keywords:** allodynia, neuropathic pain, pain, pharmacodynamics, pharmacokinetics, S1P receptor

## Abstract

**Introduction:**

Preclinical evidence suggests that dysregulation of the ceramide‐sphingosine‐1‐phosphate (S1P) axis may have analgesic properties. However, currently approved S1PR1 modulators such as fingolimod are hindered for this indication as they induce lymphopenia. The aim of the current study was to evaluate whether the novel, selective S1PR1 agonist TRV045 has analgesic effects in a validated evoked pain test battery in healthy volunteers.

**Methods:**

In this randomized, double‐blind, double‐dummy, placebo‐controlled, four‐way cross‐over study, 25 male or female healthy volunteers were randomized to receive either placebo or TRV045 50, 150 or 300 mg at separate occasions. The primary endpoint was heat pain detection threshold on UVB‐induced inflammatory pain. Secondary endpoints included analgesic reduction of capsaicin‐induced allodynia and pain detection and tolerance thresholds to evoked cold, electrical, pressure and heat pain. Furthermore, safety, tolerability and pharmacokinetics were monitored throughout the study. Data analyses included a repeated‐measures mixed‐effects model.

**Results:**

TRV045 was well tolerated, without serious adverse events. Compared to placebo, TRV045 did not reduce heat pain detection thresholds on UVB‐exposed skin, but significantly reduced the secondary area of capsaicin‐induced pain for the higher dose levels: 150 mg: −304.10 mm^2^ (95% CI: −494.78 to −113.43, *p* = 0.002); 300 mg: −298.51 mm^2^ (−486.83 to −110.20, *p* = 0.002). No effects on peripheral lymphocyte count were observed.

**Conclusion:**

TRV045 is a well‐tolerated S1PR1 modulator with analgesic properties in healthy volunteers as measured by the capsaicin‐induced pain model, which provides initial clinical evidence that selective S1PR1 modulation may be an appropriate novel target for the treatment of neuropathic pain.

**Significance Statement:**

Selective S1PR1 modulation produced analgesic effects in a validated human experimental pain model. This study provides clinical proof‐of‐concept for S1PR1 as a promising therapeutic target for neuropathic pain.

## 1 Introduction

Chronic pain is a major public health problem, affecting approximately one in five adults in Western countries (Zhu et al. 2025). Current pharmacological management is largely symptom‐driven and relies on acetaminophen, non‐steroidal anti‐inflammatory drugs (NSAIDs), opioids and adjuvant therapies such as antidepressants and antiepileptics. However, these treatments often provide insufficient analgesia and are associated with substantial adverse effects, highlighting the need for more effective and safer therapeutic strategies (Busse et al. 2018; Cohen et al. 2021; Squillace et al. 2020).

The ceramide–sphingosine‐1‐phosphate (S1P) axis has recently emerged as a potential target for pain treatment (Langeslag and Kress 2020). S1P exerts its effects through five G‐protein‐coupled receptor subtypes (S1P receptor type 1 through 5) that are widely distributed throughout the peripheral and central nervous system (Aoki et al. 2016, 2019; Trayssac et al. 2018). In preclinical models of neuropathic pain, S1P signaling is dysregulated and contributes to enhanced nociceptive processing in the spinal cord and possibly supraspinal structures (Chen et al. 2019; Salvemini et al. 2013; Squillace et al. 2020).

Pre‐clinical evidence suggests that the S1P receptor type 1 (S1PR1) might be implicated in the initiation and maintenance of (neurogenic) inflammation and mechanisms underlying peripheral and central sensitization (Benarroch 2021). Accordingly, S1P receptor modulators, such as fingolimod and ozanimod, have shown promise in experimental studies for mitigating neuropathic pain (Chen et al. 2019; Coste, Pierre, et al. 2008; Doolen et al. 2018; Sim‐Selley et al. 2009, 2018; Tran et al. 2017; Yamazaki et al. 2020; Zhang et al. 2015). However, the clinical applicability for pain treatment is limited by immunomodulatory effects, including lymphopenia (Francis et al. 2014; Tran et al. 2017).

TRV045 is a novel, orally bioavailable, S1P1 receptor modulator currently under development for the treatment of chronic pain (Chen et al. 2023; Kramer et al. 2020). In preclinical studies, TRV045 demonstrated analgesic efficacy in two neuropathic pain models. In the streptozotocin hyperglycemic model in rats, TRV045 reversed thermal hyperalgesia to an extent comparable to gabapentin. In a mouse model of paclitaxel‐induced neuropathic pain, TRV045 attenuated mechanical and cold hypersensitivity (Chen et al. 2023; Kramer et al. 2020). To date, the analgesic potential of TRV045 has not been evaluated in humans.

The aim of the present study was to explore the analgesic effects of several dose levels of TRV045 in healthy volunteers using a validated battery of evoked pain models (PainCart), which assesses multiple nociceptive mechanisms and has been used extensively to evaluate analgesic potency in the phase of early drug development (Hijma et al. 2021; Okkerse, Hay, et al. 2017; Siebenga 2020; Siebenga et al. 2018; Siebenga, van Amerongen, Okkerse, et al. 2019). Given the proposed anti‐inflammatory properties of TRV045, we hypothesized that its effects would be most pronounced in an ultraviolet‐B radiation (UVB)‐induced inflammatory pain model, designated as the primary outcome measure. This model previously showed high sensitivity for detecting analgesic effects of anti‐inflammatory agents, such as ibuprofen (Okkerse, van Amerongen, et al. 2017). Secondary outcome measures included cold, electrical, pressure and heat pain models, as well as a capsaicin‐induced model to assess primary hyperalgesia and secondary allodynia, allowing characterization of the broader analgesic profile of TRV045.

## 2 Methods

The present study was conducted at the Centre for Human Drug Research in Leiden, The Netherlands, in accordance with the Declaration of Helsinki of 1975, updated in 2013. Approval for the study was granted by the Medical Ethics Committee, Stichting Beoordeling Ethiek Biomedisch Onderzoek (BEBO) in Assen, Netherlands, and the study was prospectively registered under EudraCT number 2022‐501804‐80‐00.

### 2.1 Trial Participants

Twenty‐five healthy volunteers of either sex were enrolled to participate in the study. Prior to commencing any study evaluations, written informed consent was obtained from all participants. Inclusion criteria included men and women between 18 and 55 years of age (inclusive); body mass index between 18 and 32 kg/m^2^ (inclusive) and a body weight ≥ 50 kg at screening. Exclusion criteria included the history or presence of any medical, neurological or psychiatric disease; history of alcohol abuse, illicit drug use or any history of drug abuse or addiction; presence of abnormalities at screening or at admission to the clinical trial unit on physical examination, vital signs measurements (heart rate < 50 or > 100 beats per minute, systolic blood pressure > 140 mmHg or diastolic > 90 mmHg), electrocardiogram (QTcF > 450 msec (if male), > 470 msec (if female), PR interval > 200 msec or any cardiac dysrhythmias) or laboratory evaluations (complete blood count, kidney function and liver enzymes); pregnancy or lactation; unwillingness to use contraception from screening through 180 days after drug administration; unwillingness to ingest a high‐fat meal during dosing days, or indicating at screening to be unable to finish the meal within 30 min; Fitzpatrick skin type IV, V or VI, or wide‐spread acne, tattoos, or scarring interfering with the area of evoked pain test; intolerability to the pain tests at screening or observation that tolerance for an individual occurred at > 80% of maximum input intensity for the cold pressor or electrical pain tasks; minimal erythema dose for the UVB pain model higher than 355 mJ/cm^2^ at screening; allergy to capsaicin treatment as assessed during screening; unwillingness to comply with the requirements of the study and the inability to communicate with the study personnel effectively.

### 2.2 Study Design

**FIGURE 1:**
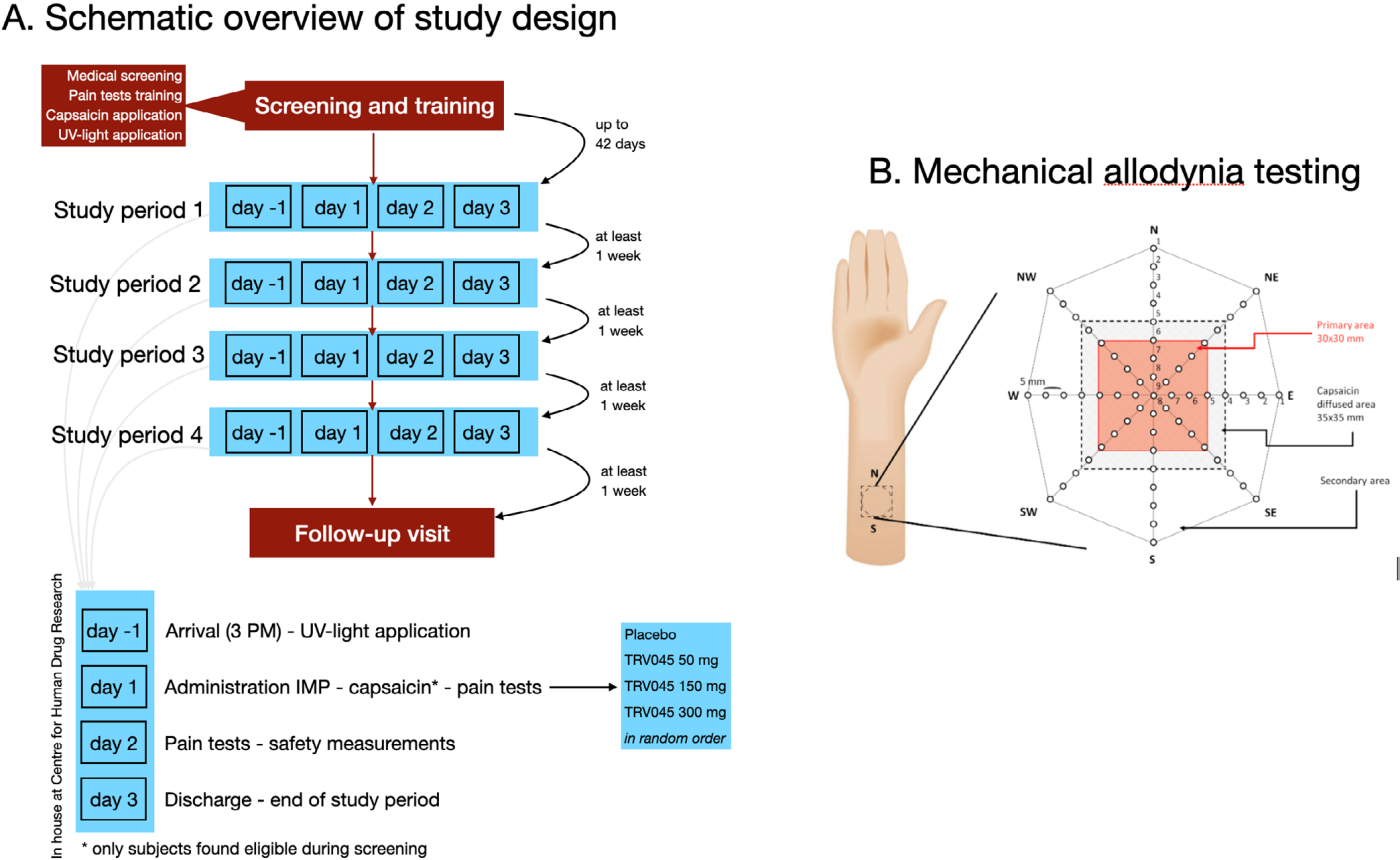
(A) Schematic overview of the study design. (B) Secondary mechanical allodynia assessment. Eight spokes, evenly dividing a circle, were marked on the volar forearm to measure the extent of secondary allodynia. The evaluation commenced at the outermost point of the primary area (#1) on the north spoke, advancing towards the center in 5 mm increments. When the sensation transitioned from almost painful to painful, that particular point was identified as the border of the allodynic region. This process was repeated for all spokes in a clockwise manner. The area of allodynia was measured in mm^2^, utilizing individual values for each spoke. To mitigate the risk of reporting false positive effects, a 5 mm border zone surrounding the primary area (i.e., the 3 × 3 cm capsaicin application area) was incorporated.

The study had a randomized, double‐blind, double‐dummy, placebo‐controlled, four‐way cross‐over design (Figure 1A). In four identical study periods, participants received either a dose of TRV045 50 mg, 150 mg, 300 mg, or placebo, in randomized order. A wash‐out period of at least 7 days separated each period. Study staff and investigators were blinded to the dose level until database lock. Block‐randomization, using Williams squares to balance for first carry‐over effects, was obtained using SAS (version 9.4, SAS Institute Inc., Cary, NC) by a statistician not involved in clinical trial management. TRV045 capsules were taken 30 min after a high‐fat meal to enhance gastrointestinal absorption.

For each study period, subjects were admitted to the research facility the day before study drug administration to perform pre‐dose physical examinations and reconfirm eligibility. The next morning baseline PK samples and pain assessments were obtained followed by study drug administration. At regular intervals after study drug administration safety assessments, PK samples (15, 30, and 45 min, and 1, 2, 4, 6, 8, 10, 24, 30 and 48 h post‐dose) and pain test (1, 2, 4, 6, 8,10, 24, 30 and 48 h after drug intake) were obtained. Subjects were discharged on Day 3 of the study period. A follow‐up visit took place 7–9 days after completion of the fourth period.

### 2.3 Treatment

TRV045 and its corresponding placebo were administered orally. The rationale for the selected doses in this study was based on scientific considerations to safely bracket a targeted dose–response range. Nonclinical toxicology studies determined a no observed adverse effect level (NOAEL) dose of 1000 mg/kg, which resulted in 3‐ to 8‐fold higher TRV045 plasma exposures than observed after administration of the highest dose tested in the first‐in‐human study, namely 300 mg given under fed conditions. Previous phase 1 studies demonstrated that this dose was well‐tolerated. Furthermore, this 300 mg dose in fed subjects yielded unbound maximum plasma concentrations (C_MAX_) that exceeded the in vitro half‐maximal effective concentration (EC_50_) for TRV045‐induced stimulation of S1PR1 G protein signaling of approximately 32 nM (equivalent to an estimated free plasma concentration of 10.8 ng/mL). Preclinical animal models of neuropathic pain in rats demonstrated analgesic effects at a dose that led to an estimated free drug concentration of 6 ng/mL (Kramer et al. 2020). The chosen doses for this study, 50, 150 and 300 mg (administered to earlier fed subjects), were therefore selected to encompass a spectrum of exposures for the assessment of the pharmacokinetic and pharmacodynamic relationship. The Phase 1, first‐in‐human study demonstrated a modest food effect, where fed conditions (i.e., a high fat meal) provided the highest plasma exposure to oral administration of TRV045.

### 2.4 Pain Tests

A comprehensive and validated battery of evoked pain tests was used to determine the analgesic effects induced by TRV045 and placebo (Hijma, Moss, et al. 2022; Siebenga, van Amerongen, Okkerse, et al. 2019). Applied evoked pain tests included: (1) heat pain detection thresholds on UVB skin (primary outcome), on capsaicin treated skin (primary hyperalgesia), and on normal skin; (2) mechanical allodynia after capsaicin application (total and secondary allodynia); (3) cold pressor pain and tolerance thresholds; (4) electrical pain detection and tolerance thresholds (stair and burst paradigm); and (5) pressure pain and tolerance thresholds (Hijma, Moss, et al. 2022; Siebenga, van Amerongen, Okkerse, et al. 2019). Here, we describe the different pain tests in short. For more extensive details on all pain tests, see the Supporting Information.

To induce the UVB pain model, the minimal erythema dose (MED) was determined for each subject. To that end, six doses of UVB irradiation were applied to 1 cm^2^ areas on the upper back, with doses varying from 64 to 1321 mJ/cm^2^ based on the average MED for different skin phototypes. After 18–24 h, the MED was visually identified as the lowest UVB dose causing visible erythema. Next, during the experiment days, twice the subject's UVB MED (2MED) was applied to a 3‐cm^2^ area on the right scapula and heat pain detection thresholds (applied with a 3 × 3 cm heat probe) were determined on the UV‐B irradiated area. Pain was quantified using an electronic visual analogue scale (eVAS; ranging from 0 = no pain to 100 = worst pain imaginable), by sliding a button, ranging from 0 for ‘no pain’ to 100 for ‘worst tolerable pain.’ The pain detection threshold was quantified as the temperature at which the participants started to feel the heat stimulus as painful. Secondary hyperalgesia following UVB‐induced inflammation was excluded from the current study due to its limited spatial extent and poor reproducibility observed during internal validation. Consequently, this model was deemed insufficiently robust for inclusion in a Phase 1 clinical trial battery designed to evaluate analgesic efficacy.

To induce mechanical allodynia, a capsaicin 1% ethanolic solution was applied to a 3 × 3 cm area on the dominant volar forearm 60 min before study drug administration (t = 0). Thirty minutes after the application, the excess of capsaicin was wiped towards the center of the application site and removed from the skin. The total and secondary allodynia area was determined using Von Frey hair filaments. Before drug dosing, filaments of 128, 256 and 512 mN were applied outside the stimulated area, and participants identified the filament that felt “nearly painful” for use during testing. A 9‐cm^2^ square primary area was mapped over the capsaicin treated area (primary area) with eight evenly spaced spokes (45° apart), dividing it into an octagon. Along each spoke, nine stimulation points (5 mm apart) were designated; see Figure 1B. Stimulation proceeded from the outermost point inward, and once a participant reported a change from ‘nearly painful’ to ‘painful’, that point was marked as the boundary of the primary or secondary allodynia area (within or outside the capsaicin treated area). Testing continued in a clockwise fashion around the octagon. To prevent false‐positive measurements, a 5 mm border around the primary area was left untested (Mohammadian et al. 1998; Torebjörk et al. 1992).

For the cold, electrical and pressure pain tests, two thresholds were recorded for each test: the pain detection threshold (PDT), which refers to the point where subjects start to feel pain, and the pain tolerance threshold (PTT), which refers to the point where pain became intolerable. In short, cold pain was induced by immersion of the subject's nondominant hand into cold water. First, the subject's hand was placed in a circulating water bath with a temperature of 35°C ± 0.5°C for 2 min. After 1 min and 45 s, a blood pressure cuff was inflated on the upper arm to restrict blood flow followed by (at the 2‐min mark) a transfer of the subject's hand into a cold‐water bath with a temperature of 1.0°C ± 0.5°C. For the electrical pain test, two Ag‐AgCl electrodes were placed on clean skin over the left tibial bone. To induce pain, a current was applied between the electrodes with incremental increases of the current intensity by 0.5 mA per second. The difference between the electrical stair and burst tests is that the stair test is a single increasing stimulus that induces electrical pain detection and tolerance thresholds via activation of the sensory nerves while bypassing the nociceptors in the skin. In contrast, the burst test includes a repetitive electrical stimulus and is used as a proxy of windup as a marker for pain facilitation (Arendt‐Nielsen and Yarnitsky 2009; Olofsen et al. 2005). Pressure pain was applied to the gastrocnemius muscle using an 11‐cm‐wide tourniquet cuff. To increase pressure on the muscle, the cuff was inflated at a rate of 0.5 kPa/s, which induces a deeper muscle pain.

Pain tests were conducted in fixed order: allodynia testing on the dominant volar forearm → heat pain tests on the dominant volar forearm (untreated and capsaicin treated skin) → heat pain tests on the upper back (normal skin and UVB treated skin) → pressure pain test on the lower leg → electrical burst test on the skin overlying the left tibial bone—cold pressor test on the non‐dominant hand—electrical stair test on the skin overlying the left tibial bone. The combination of an electrical stair test, a cold pressor test, and a second electrical stair test is used to assess conditioned pain modulation (CPM). In previous validation studies no cross‐over effects were observed for fixed order testing (Hijma, Moss, et al. 2022; Siebenga, van Amerongen, Klaassen, et al. 2019; Siebenga, van Amerongen, Okkerse, et al. 2019).

### 2.5 Blood Sampling

Venous blood samples (5 mL) were collected in K2‐EDTA tubes, centrifuged and stored at −80°C until analysis. Plasma TRV045 concentrations were analyzed by Frontage (Exton, PA, USA) using validated high‐performance liquid chromatography with tandem mass spectrometry. The lower limit of quantitation was 1 ng/mL, with an assay coefficient of variation below 15%.

### 2.6 Safety

Safety assessments, including hematology analyses (complete blood count), clinical chemistry (kidney function and liver enzymes) and vital signs, 12‐lead electrocardiograms (ECGs), and adverse event monitoring, were carried out throughout the study. Adverse events were any event reported by the subject at any timepoint after first admission or abnormal values of laboratory tests, vital signs, or ECGs. Treatment‐emergent adverse events (TEAEs) were defined as an adverse event (AE) observed after starting the administration of the treatments up to 7 days after study drug administration. All TEAEs were coded according to the Medical Dictionary for Regulatory Activities (MedDRA), version 25.

Since peripheral lymphopenia is a known on‐target effect of non‐selective S1PR1 modulators, peripheral lymphocyte count was a laboratory parameter of special interest. Subjects with baseline values for lymphocytes below the normal laboratory reference range (1.0 to 3.5 × 10^9^ cells/L) were excluded from analysis, given that these values could underestimate possible lymphopenia caused by TRV045.

### 2.7 Statistical Analysis

In designing this exploratory study, we based our sample size on UVB heat pain detection threshold data because of its well‐described effects on the inflammatory pain response in prior studies and its link to the proposed mechanism of action of TRV‐045. A sample size of 24 was estimated to have a power of 0.80 to detect a mean difference in UVB heat pain detection test of 2.1% (two‐sided test) between treatments, assuming a standard deviation of differences of 0.035 and alpha = 0.05 (Siebenga, van Amerongen, Klaassen, et al. 2019).

All statistical analyses were conducted in the modified intention‐to‐treat population, defined as all randomized participants who received at least one dose of study medication and had at least one post‐baseline pharmacokinetic and pharmacodynamic measurement. Pharmacokinetic data were analyzed using a non‐compartmental approach. All measured pharmacodynamic endpoints were analyzed with a mixed model analysis of covariance. This modeling approach was chosen to account for the crossover and repeated‐measures design of the study, in which each participant received multiple treatments across different periods and contributed repeated measurements over time. Because observations within individuals are correlated, a mixed‐effects framework was used to appropriately model intra‐individual correlation and between‐subject variability. Treatment, time, period, and treatment‐by‐time interaction were included as fixed effects. Participant and participant‐by‐treatment and participant‐by‐time interaction terms were included as random effects to account for individual variability in treatment response and temporal patterns. The (average) baseline measurement per period was included as a covariate. The overall treatment effect (TRV045 versus placebo) and specific contrasts per dose (TRV045 50 mg versus placebo, TRV045 150 mg versus placebo, and TRV045 300 mg versus placebo) were reported with the estimated difference and the 95% confidence interval (CI), the least square mean (LSM) estimates, and the *p*‐value. All analyses were made with a significance threshold of 95%. A Bonferroni correction to control for a potential type I error arising from multiple comparisons was applied post hoc. As three specific contrasts were tested in this study and 8 different pain tests were evaluated, the adjusted significance threshold was set at *p* = 0.05/24 = 0.0021.

## 3 Results

### 3.1 Subjects

**FIGURE 2:**
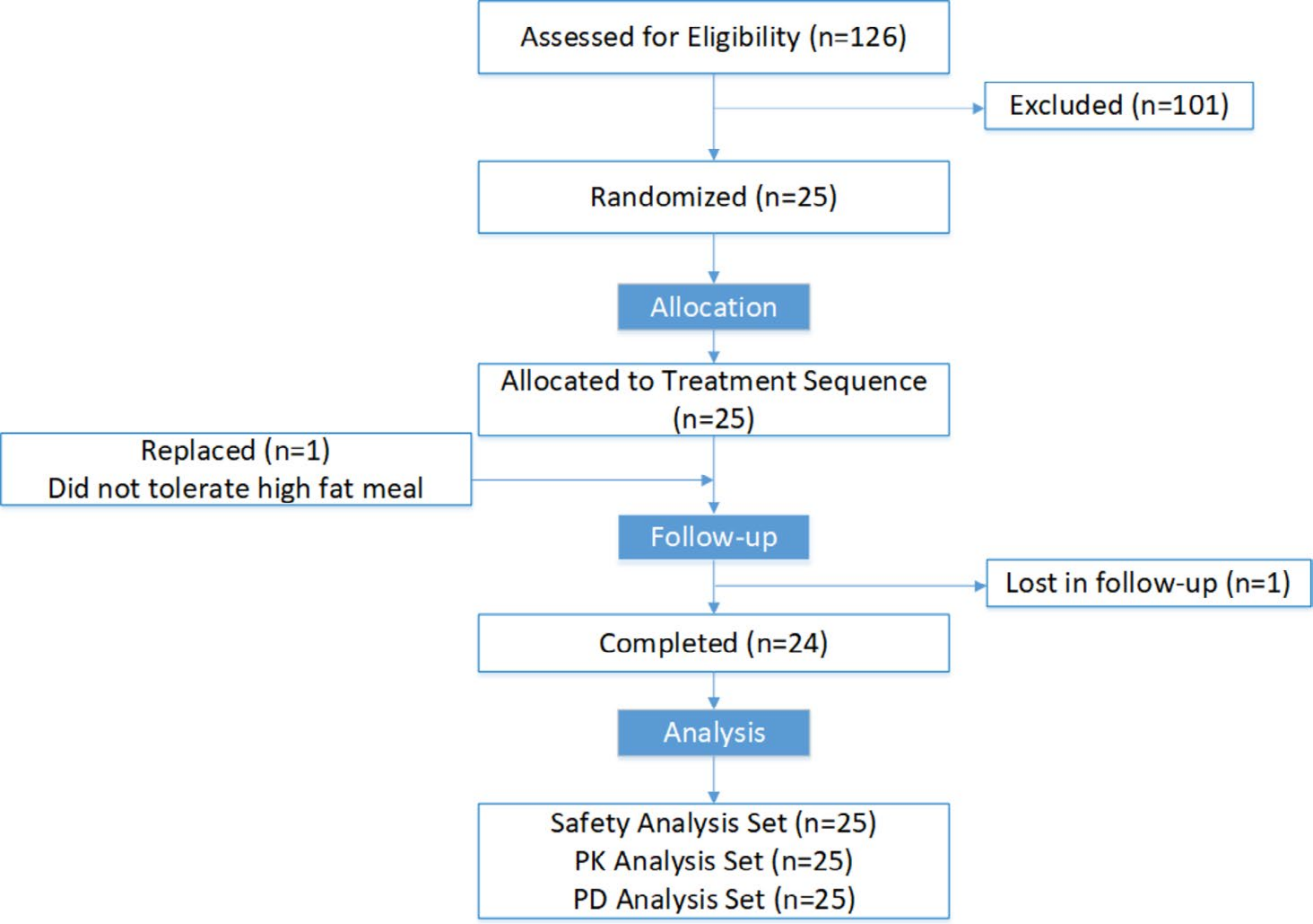
CONSORT flow diagram.

A total of 25 participants were enrolled and randomized to the treatment regimens (Figure 2). One participant was replaced prior to dosing because he was unable to consume the required high‐fat meal at the first visit and therefore did not receive study medication. Twenty‐four participants received study treatment and completed all study periods. One participant withdrew after completion of the final study period but before the follow‐up visit for personal reasons. The statistical analyses were performed in the modified intention‐to‐treat population, comprising all 24 participants who received at least one dose of study medication and had post‐baseline pharmacodynamic assessments. All available data were included in the analyses, except for lymphocyte data from two participants, which were excluded due to baseline lymphocyte counts below the normal laboratory reference range (1.0–3.5 × 10^9^ cells/L). Baseline characteristics of the participants are presented in Table 1.

**TABLE 1:** Demographics of the subject population (*n* = 25).

Age (years)	38 ± 11 (18–53)
Height (cm)	178 ± 10 (157–193)
Weight (kg)	76 ± 13 (51–107)
Body mass index (kg/m^2^)	24 ± 3 (19–31)
Sex ratio—male/female	17/8 (68%/32%)

*Note:* Data are mean ± SD (range); for sex ratio data are *n* (%).

### 3.2 Pharmacokinetics

**FIGURE 3:**
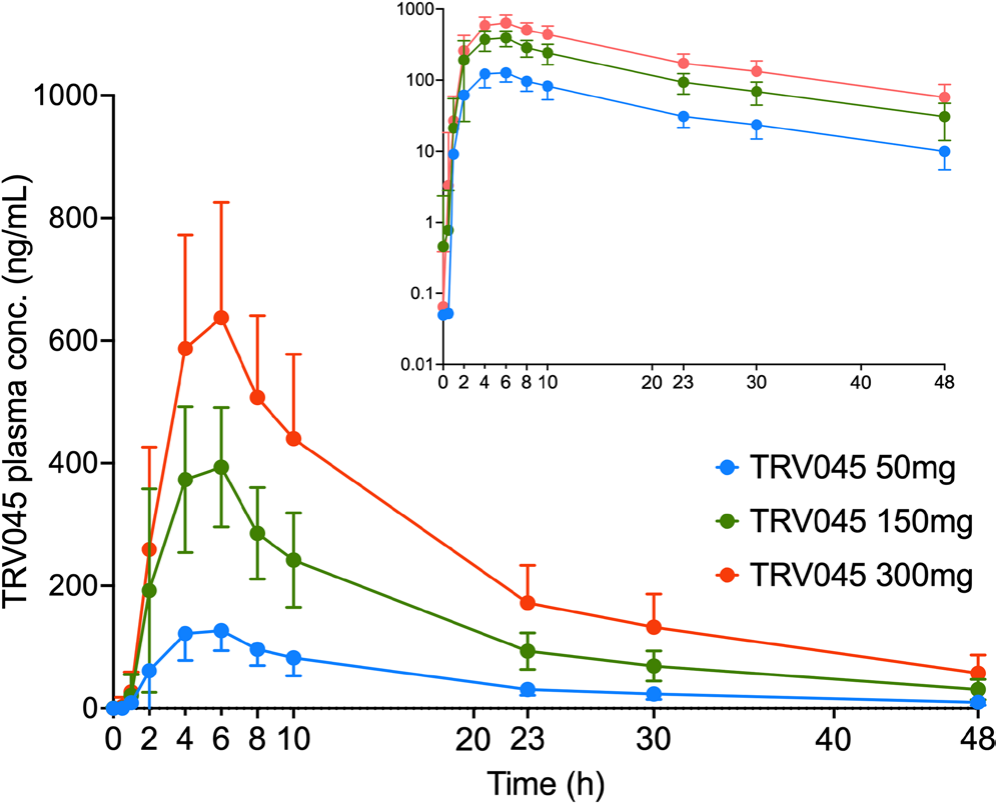
Plasma concentration of TRV045 following dosing with 50, 150 and 300 mg. Data are mean ± SD. The inserted box at the right top corner represents the log‐transformed plasma concentrations.

In Figure 3, the plasma concentrations of the 3 TRV045 doses are given. Mean plasma concentrations increased over time and peaked at approximately 6 h for all three dose levels, with peak concentrations of 149.8 ± 30.19 ng/mL (50 mg), 446.8 ± 105.69 ng/mL (150 mg), and 682.9 ± 182.22 ng/mL (300 mg). The exposures of TRV045 showed peak concentrations at T_MAX_ of 6 h, with an apparent half‐life, t½, of 14.7 ± 3.5 h (50 mg), 15.0 ± 5.1 h (150 mg), and 14.7 ± 4.1 h (300 mg) for the different TRV045 dose levels studied. There was a linear increase in maximum concentration (C_MAX_) and area under the curve to infinity (AUC_INF_) with increasing doses for the TRV045 50 and 150 mg dose levels but not for the 300 mg dose that showed lower plasma concentrations than expected by linear extrapolation (Figure 3). See also Table S1 for all parameter values of the non‐compartmental analysis.

### 3.3 Evoked Pain Tests

The results of all pain tests for the three TRV045 dose levels compared to placebo are listed in Table S2.

#### 3.3.1 UVB‐Pain Model

TRV045 did not significantly change heat pain detection thresholds at any TRV045 dose levels compared to placebo (Table S2). The estimate of difference for 50 mg TRV045 versus placebo was 0.31°C with a 95% CI of −0.20 to 0.82 (*p* = 0.231), for 150 mg TRV045 versus placebo 0.05°C with a 95% CI of −0.64 to 0.57 (*p* = 0.836) and 0.31°C with a 95% CI of −0.20 to 0.83 (*p* = 0.229) for 300 mg TRV045 versus placebo.

#### 3.3.2 Total (Primary Plus Secondary) Area of Mechanical Allodynia

**FIGURE 4:**
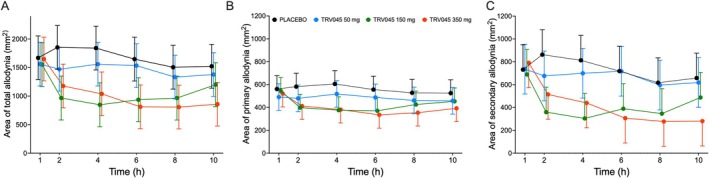
Effect of TRV045 and placebo on total (A), primary (B) and secondary (C) areas of allodynia. TRV045 at 150 and 300 mg had greater reduction of total (*p* < 0.001) and secondary (*p* = 0.002) areas of allodynia versus placebo. Values are mean ± SD.

TRV045 significantly decreased the total area of allodynia at 150 and 300 mg dose levels compared to placebo (Figure 4). The estimate of the difference for 150 mg TRV045 versus placebo was −595.3 mm^2^ with a 95% CI of −902.0 to −288.6 (*p* = 0.0002), and for 300 mg TRV045 versu*s* placebo −616.4 mm^2^ with a 95% CI of −917.0 to −315.8 (*p* < 0.001).

#### 3.3.3 Secondary Mechanical Allodynia

Compared to placebo, TRV045 significantly decreased the area of secondary allodynia at 150 and 300 mg (Figure 4). The estimate of the difference for 150 mg versus placebo was −304.1 mm^2^ with a 95% CI of −494.8 to −113.4 mm^2^ (*p* = 0.002) and for 300 mg −298.5 mm^2^ with a 95% CI of −486.8 to −110.2 (p = 0.002).

#### 3.3.4 Other Evoked Pain Models

No significant effects were detected for the pain detection and tolerance thresholds for the cold pressor test, the electrical pain tests, the pain pressure test, and the heat pain test on normal and capsaicin‐exposed skin. See Table S2 for the estimated mean differences of the different dose levels of TRV045 compared to placebo.

### 3.4 Safety

A summary of treatment‐emergent adverse events is provided in Table S3. There were no serious adverse events during the study. The AEs in all participants were mild apart from 1 participant treated with TRV045 150 mg who had 1 moderate AE (epistaxis) and 1 participant treated with TRV045 300 mg who also had 1 moderate AE (viral infection). All the reported AEs were transient and resolving without any subsequent complications, subject withdrawal, or the need for interventions. Clinical laboratory parameters, electrocardiogram (ECG), and vital signs revealed no clinically significant changes during the study.

### 3.5 Lymphocyte Counts

No clinically meaningful change in lymphocyte count, or other safety findings related to lymphopenia were observed in any subject after administration of any of the doses of TRV045. Lymphocyte data from two subjects were excluded from analyses based on baseline blood lymphocyte values below the normal laboratory reference range of 1.0 to 3.5 × 10^9^ cells/L. One subject was excluded from the analysis of the TRV045 300 mg treatment group (baseline lymphocyte value: 0.75 × 10^9^/L), another from analyses of all treatment groups (0.86 × 10^9^/L (placebo), 0.76 × 10^9^/L (50 mg), 0.81 × 10^9^/L (150 mg) and 0.81 × 10^9^/L (300 mg)).

**FIGURE 5:**
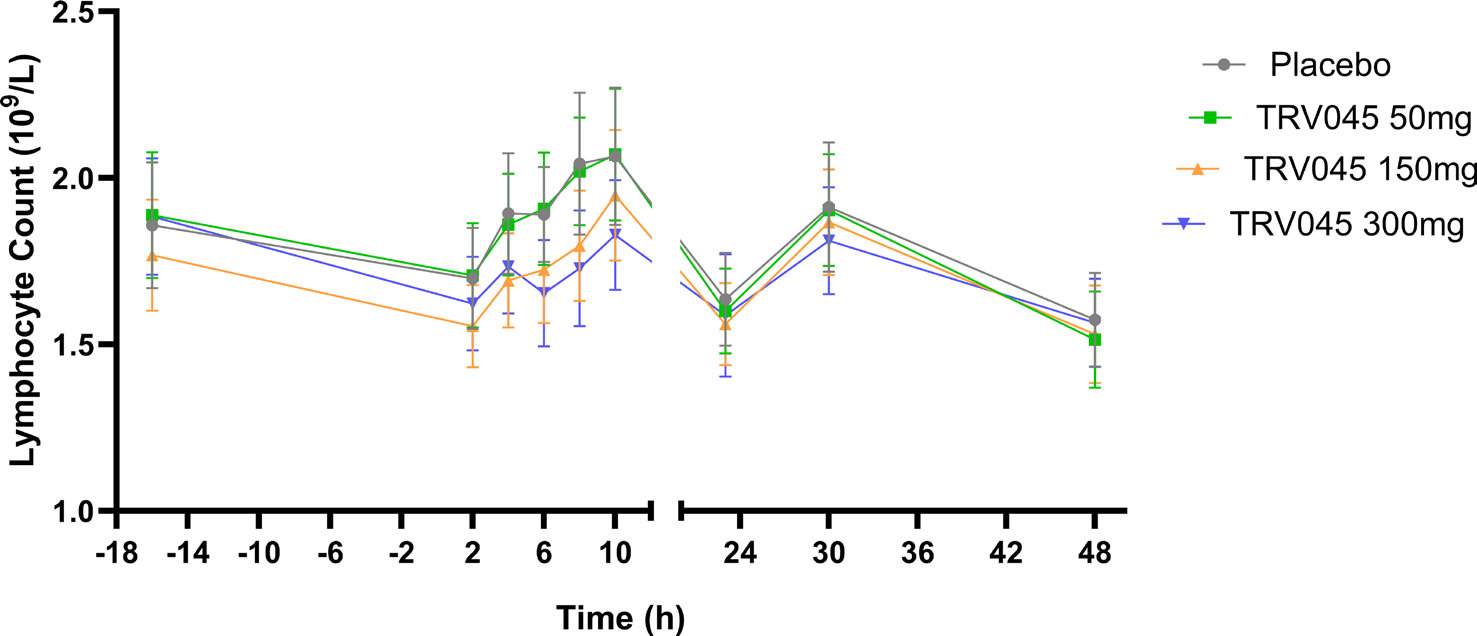
Influence of placebo and 50, 150 and 300 mg TRV045 on lymphocyte count. Data are mean ± 95% CI.

Figure 5 gives the lymphocyte count for those subjects who had lymphocyte counts within the normal laboratory reference range at all time points prior to exposure to study drug or placebo. Among the subjects, five had isolated total lymphocyte counts at a single observation time point that fell below the laboratory normal lower range of 1.0 × 10^9^ cells/L. Two subjects dosed with 50 mg had blood lymphocyte values of 0.95 × 10^9^/L (Day 2) and 0.96 × 10^9^/L (Day 3); two other subjects dosed with 150 mg had values of 0.97 × 10^9^/L (Day 2) and 0.83 × 10^9^/L (Day 3); and finally, one subject dosed with 300 mg had blood lymphocyte values of 0.97 × 10^9^/L (Day 1). For these subjects, blood lymphocyte values at all other timepoint were within the normal laboratory reference.

## 4 Discussion

In this exploratory study, we applied a battery of evoked nociceptive tests (PainCart) to characterize the analgesic profile of TRV045 in healthy volunteers, a novel drug candidate being developed for the treatment of chronic pain. TRV045 did not show analgesic effects on the UVB induced inflammatory (primary) hyperalgesia (primary endpoint). Still, TRV045 significantly reduced both the secondary and total area of mechanical allodynia in the capsaicin‐induced sensitization model compared to placebo. Furthermore, TRV045 was safe and well tolerated and did not induce lymphopenia after a single administration. Although these results should be interpreted cautiously given the exploratory nature of the study, they provide preliminary evidence that selective S1PR1 modulation may influence specific nociceptive pathways in humans.

### 4.1 The Nociceptive Test Battery

This study is the first to evaluate the analgesic effects of a selective S1PR1 modulator in humans using a battery of experimental evoked pain models. For this, a validated battery of pain tests known as PainCart was performed, which has previously shown sensitivity to compounds with distinct mechanisms of action with high within‐day reliability and reproducibility (Okkerse, Hay, et al. 2017; Siebenga et al. 2018; Siebenga, van Amerongen, Okkerse, et al. 2019). For example, sodium channel blockers, including precursors of the recently approved agent suzetrigine, demonstrated pronounced effects in the cold pressor test (Hijma et al. 2021; Hijma, Moss, et al. 2022). Based on the proposed anti‐inflammatory properties of TRV045, we hypothesized that the compound would exert measurable effects in the UVB‐induced inflammatory hyperalgesia, as non‐steroidal anti‐inflammatory drugs such as ibuprofen have shown sensitivity in this paradigm (Okkerse, van Amerongen, et al. 2017). Contrary to our expectations, TRV045 did not affect UVB heat pain thresholds, suggesting that acute inflammatory nociceptive processing in this model may not be substantially modulated by selective S1PR1 antagonism under the conditions tested.

Among the secondary endpoints, a significant effect was observed only in the capsaicin‐induced sensitization model, where TRV045 reduced the area of secondary mechanical allodynia without affecting primary hyperalgesia. Capsaicin activates TRPV1 receptors on C‐ and Aδ‐fiber nociceptors, inducing a primary hyperalgesic area at the application site and a surrounding secondary allodynic area that is generally attributed to central sensitization mechanisms involving dorsal horn neurons and potentially supraspinal processing (Messeguer et al. 2006; Mohammadian et al. 1998; Torebjörk et al. 1992). The selective reduction of secondary allodynia only might indicate that the effects of S1P rely on modulation of central sensitization processes; however, further research is warranted to further explore this hypothesis. Preclinical data demonstrating involvement of S1P signaling in spinal nociceptive processing suggest involvement of central processes (Chen et al. 2023; Kramer et al. 2020; Warwick et al. 2022). However, a peripheral contribution cannot be excluded, as S1P has been shown to interact with TRPV1‐mediated hypersensitivity in animal models, and attenuation of such responses has been reported in TRPV1‐deficient mice (Squillace et al. 2020; Torebjörk et al. 1992; Trayssac et al. 2018).

The absence of effects in other experimental pain modalities, including electrical, heat, and pressure stimulation, suggests that TRV045 does not broadly alter acute nociceptive thresholds in healthy volunteers, which also was not hypothesized based on preclinical studies. Differential sensitivity across evoked pain models is not uncommon in early analgesic development. For instance, sodium channel blockers typically demonstrate relative sensitivity to cold pressor pain, whereas anti‐inflammatory agents show selective effectiveness on UVB‐induced inflammatory hyperalgesia, while not affecting other nociceptive paradigms (Hijma, van Brummelen, et al. 2022; Hijma and Groeneveld 2021; Okkerse, van Amerongen, et al. 2017). The inclusion of multiple mechanistically distinct nociceptive models in phase 1 trials enables comprehensive pharmacodynamic profiling and supports informed decision‐making in early clinical development (Hijma et al. 2021; Siebenga, van Amerongen, Okkerse, et al. 2019). Whether the observed modulation of capsaicin‐induced allodynia translates into clinically meaningful analgesic effects in chronic pain populations remains to be established in subsequent studies.

### 4.2 TRV045 Mechanism of Action

The mechanism by which S1PR1 activation results in anti‐nociceptive effects has not been fully elucidated. Analgesic effects of a different S1PR1 modulator, fingolimod, have been attributed to both its agonist signaling (Coste, Brenneis, et al. 2008; Coste, Pierre, et al. 2008; Doolen et al. 2018; Sim‐Selley et al. 2009, 2018) or to functional antagonism (Mohammadian et al. 1998; Torebjörk et al. 1992). Within the neuro‐immune coupling framework, fingolimod‐mediated modulation of S1PR1—likely via functional antagonism (Chen et al. 2019; Salvemini et al. 2013)—is proposed to suppress astrocyte activation, leading to reduced expression of pro‐inflammatory cytokines (IL‐6, TNFα, IL‐1β) and a concomitant increase in the anti‐inflammatory cytokine IL‐10. The subsequent inhibition of glutamate release on primary afferent terminals induces pain relief (Squillace et al. 2020).

However, the literature on the effects of S1PR1 regulation on glutamate is conflicting. For example, fingolimod has been shown to both inhibit and potentiate glutamate release through activation of S1P receptors (Darios et al. 2017; Doyle et al. 2020). Endogenous S1P, through engagement of its various receptors, is thought to be pronociceptive. It is likely that the effects of exogenous drugs targeting S1P receptors, for example on glutamate release, need to be considered in the context of coordinated actions within the endogenous S1P system, likely resulting in complex and site‐specific interactive effects. Additionally, other mechanisms may be involved in the analgesic effects of S1PR1 agonism, such as S1PR1‐induced reduction of prostaglandin synthesis through the inhibition of cytosolic phospholipase A2 (Coste, Brenneis, et al. 2008). Overall, it seems that CNS S1P receptors are part of a widespread neuromodulatory system and that the S1PR1 contributes to S1P‐mediated antinociception. Whether TRV045 has a primarily agonistic signaling effect or acts as a functional antagonist requires further study. Recent evidence suggests that TRV045 exhibits distinct pharmacological properties from the S1P modulator fingolimod, with different effects on receptor desensitization and downregulation in CNS regions involved in pain perception (Selley et al. 2024). These data suggest that TRV045 acts as an agonist at the S1P site with downstream activation of antinociceptive pathways (Squillace et al. 2020; Torebjörk et al. 1992).

### 4.3 Lack of Lymphopenia

Sustained peripheral lymphopenia is a common on‐target effect of other S1PR1 modulating drugs like fingolimod (Ohtani et al. 2018). In the current study, after single administration, the lack of peripheral lymphopenia suggests that TRV045 might have on‐target actions distinct from those of fingolimod, with limited impact on lymphocyte trafficking in peripheral tissues. Such differentiation may be operative in the central nervous system, where, in contrast to TRV045, fingolimod causes S1PR1 desensitization and down‐regulation (Selley et al. 2024). The effect of TRV045 on lymphocytes after multiple administrations is currently unknown.

### 4.4 Strengths and Limitations

The use of a comprehensive battery of evoked pain models in this phase 1 study can be considered both a strength and a limitation. While the inclusion of multiple endpoints may raise concerns regarding multiplicity and exploratory testing, such an approach is common in early‐phase analgesic drug development due to its inherent exploratory character (Kalliomäki et al. 2012; Turk et al. 2008). Experimental pain models are specifically designed to explore pharmacodynamic effects, provide preliminary evidence of target engagement and mechanism of action, and support informed go/no‐go decisions before advancing to larger and more costly patient trials (Hijma and Groeneveld 2021). The inclusion of mechanistically distinct models allows for characterization of a compound's analgesic profile across different nociceptive pathways. The analgesic signal observed for TRV045 in the capsaicin‐induced sensitization model is considered meaningful within this exploratory framework for several reasons. First, the time course of the pharmacodynamic effect was consistent with the pharmacokinetic profile of TRV045. Second, the pharmacodynamic effect that was observed was dose dependent, which is typical of true pharmacological effects. Third, the findings are in line with preclinical data supporting modulation of sensitization mechanisms. Also, the reduction in secondary allodynia remained statistically significant after correction for multiple testing. Nevertheless, the results should be interpreted with caution. The study was conducted in healthy volunteers rather than patients with chronic pain, and effects observed in evoked pain paradigms cannot be directly extrapolated to spontaneous or ongoing pain in clinical populations. Confirmation of these findings in patient‐based studies is therefore required to determine the clinical relevance of S1PR1 modulation for chronic pain treatment. Another limitation of the study is the short observation window of the effect of TRV045 on lymphocyte count. We are not informed on the effect of TRV045 with longer administration.

## 5 Conclusion

In conclusion, single‐dose administration of the selective S1PR1 modulator TRV045 significantly reduced the area of secondary mechanical allodynia in the capsaicin‐induced sensitization model, suggesting modulation of sensitization‐related nociceptive mechanisms. The compound was safe and well tolerated and did not induce lymphopenia. Although these findings are exploratory and derived from an experimental pain setting in healthy participants, they provide initial clinical evidence that selective S1PR1 modulation may influence specific components of nociceptive processing in humans. Further studies in patient populations are warranted to determine the clinical relevance of these findings for chronic pain treatment.

## Author Contributions

W.A.B., H.J.H., J.K., M.A.D., R.C., M.N., A.D. and G.J.G. wrote the manuscript. W.A.B., H.J.H., J.K., M.A.D. and G.J.G. designed the research; W.A.B., and P.E. performed the research. W.A.B., H.J.H., E.S.K., M.N., A.D. and G.J.G. analyzed the data.

## Funding

Trevena Inc., 955 Chesterbrook Blvd., Suite 110, Chesterbrook, PA 19087, United States of America was the sponsor of the study.

## Conflicts of Interest

W.A.B., P.E., E.K., H.J.H., M.N., A.D. and G.J.G. are employees of the Centre for Human Drug Research. J.K. R.C., and M.A.D. are employees of Trevena Inc.

## Supporting information


**Table S1:** Pharmacokinetic parameters of TRV045 in plasma following oral administration.
**Table S2:** Results of evoked pain tests in all treatment groups.
**Table S3:** Adverse events by medical dictionary for regulatory activities (MedDRA) system organ class and preferred term (number [%]).
