# Supplementary material for: Effects of Selective Sphingosine‐1‐Phosphate Receptor 1 Agonist, TRV045, on Evoked Pain Tests: An Exploratory, Four‐Way Cross‐Over Study in Healthy Volunteers
Source: Eur J Pain. 2026 Jun 17;30(6):e70314. doi: 10.1002/ejp.70314 (PMC13276020; doi:10.1002/ejp.70314)
Supplement: Supplementary file 1 — Table S1: Pharmacokinetic parameters of TRV045 in plasma following oral administration. Table S2: Results of evoked pain tests in all treatment groups. Table S3: Adverse events by medical dictionary for regulatory activities (MedDRA) system organ class and preferred term (number [%]). [file EJP-30-0-s001.docx]

**Supplemental materials to**

**Effects of selective sphingosine-1-phosphate receptor 1 agonist, TRV045, on evoked pain tests: an exploratory, four-way cross-over study in healthy volunteers**

Wouter A. Bakker,^1^ P. Eijsvogel,^1,2^ Erica S. Klaassen,^1^ Jessica Kim,^3^ Ruihua Chen,^3^ Mark A. Demitrack,^3^ Albert Dahan,^1^ Marieke Niesters,^1,4^ Hemme J. Hijma,^1,2^ Geert Jan Groeneveld^1,2^

1. Centre for Human Drug Research, Leiden, the Netherlands; 2. Leiden University Medical Centre, Leiden, The Netherlands; 3. Trevena Inc., Chesterbrook, PA 19087, USA; 4. Department of Anesthesiology and Pain Medicine, Erasmus Medical Center, Rotterdam, the Netherlands.

**Contents**

1. Description of evoked pain tests
2. Supplemental Table 1. Results of the non-compartmental pharmacokinetic analysis
3. Supplemental Table 2. Results of the statistical analysis of evoked pain test
4. Supplemental Table 3. Adverse events by Medical Dictionary for Regulatory Activities (MedDRA) system organ class and preferred term (number (%)).

**Description of evoked pain tests.**

**UVB Skin exposure**: During the screening visit, UVB irradiation was administered to determine each subject’s minimal erythema dose (MED). Six doses were applied to 1 cm² areas on the upper back, with doses varying from 64 to 1,321 mJ/cm² based on the average MED for different skin phototypes. After 18–24 hours, the MED was visually identified as the lowest UVB dose causing visible erythema. In the study, twice the subject's UVB MED (2MED) was applied to a 3 cm² area on the right scapula prior to the first pain task.

**Heat Pain Test:** Heat pain detection thresholds (PDTs) were assessed on skin exposed capsaicin and UVB as well as on non-exposed skin as a control area. Het pain was applied using a 3x3 heat probe of which the temperature gradually increased from 32°C at a rate of 0.5°C/s. The subjects were asked to indicate the temperature at which the heat exposures were starting to feel painful. This temperature was marked as the pain detection threshold. The test was stopped once the pain detection threshold was reached or if the temperature of the probe 50°C. The average of three measurements was used for analysis.

**Pressure Pain Test**: Pressure pain was applied to the gastrocnemius muscle at an increasing rate of 0.5 kPa/s using an 11-cm-wide tourniquet cuff (VBM Medizintechnik GmbH, Sulz, Germany). The pressure was controlled by an electro-pneumatic regulator (ITV1030-31F2N3-Q, SMC Corporation, Tokyo, Japan), an analog-to-digital converter (Power1401mkII), and Spike2 software (CED, Cambridge, UK). Subjects were asked to indicate when the pressure stimulus was starting to feel painful (pain detection threshold) and when the maximum tolerant level was reached (pain tolerance threshold). The test ended when the pain tolerance threshold was reached or at maximum pressure of 100 kPa.

**Electrical Stimulation Test**: Two Ag-AgCl electrodes were placed on clean skin over the left tibial bone, one positioned 100 mm below the patella and the other 135 mm below the first electrode. The resistance between the electrodes was maintained below 2 kΩ. In the first electrical test (stair paradigm), single electrical pulses were delivered at a frequency of 10 Hz with a duration of 0.2 ms, controlled by a computer-operated constant current stimulator. For the second electrical test (burst paradigm), a train of five 1 ms square wave pulses repeated at 200 Hz were delivered five times at a frequency of 2 Hz, with a random interval of 3 to 8 seconds between repetitions. For both test the current intensity increased by 0.5 mA per second. Pain intensity was measured using an electronic visual analogue scale (eVAS) reaching from 0 to 100 mm where 0 indicated “no pain” and 100 mm “most intense pain imaginable”. Subjects were asked to rate pain intensity using the slider on the eVAS to determine the pain detection threshold (level of first pain detected) and the electrical current evoking the most pain imaginable (pain tolerance threshold). The test ended when the pain tolerance threshold was reached or at the maximum intensity of 50 mA.

**Cold Pressor Test**: Cold pain was assessed by submerging the subject's nondominant hand into cold water. First, the subject’s hand was placed in a circulating water bath with a temperature of 35 ± 0.5°C for 2 minutes. After 1 minute and 45 seconds, a blood pressure cuff was inflated on the upper arm to restrict blood flow followed by (at the 2 minute mark) a transfer of the subject’s hand into a cold-water bath with a temperature of 1.0 ± 0.5°C. The subject was asked to indicate pain intensity using the electronic visual analogue scale (eVAS) slider to asses the time to pain detection threshold and time to pain tolerance threshold. The test ended when the PTT was reached or after 120 seconds in cold water, and the blood pressure cuff was deflated.

**Supplemental Table 1. Pharmacokinetic parameters of TRV045 in plasma following oral administration**

| **Treatment** | **Parameter** | **Mean** | **Geometric Mean** | **SD** | **CV (%)** | **Geometric CV (%)** | **Median** | **Min** | **Max** |
| --- | --- | --- | --- | --- | --- | --- | --- | --- | --- |
| TRV045 50 mg | AUC_INF_ (h*ng/mL) | 2250.1 | 2188.6 | 536.6 | 23.8 | 24.6 | 2126.3 | 1303.2 | 3464.8 |
|  | AUC_LAST_ (h*ng/mL) | 2022.2 | 1977.9 | 429.3 | 21.2 | 21.9 | 1912.3 | 1236.4 | 2806.3 |
|  | CL/F (L/h) | 23.5 | 22.9 | 5.8 | 24.8 | 24.6 | 23.5 | 14.4 | 38.4 |
|  | C_MAX_ (ng/mL) | 149.8 | 146.7 | 30.2 | 20.2 | 21.3 | 150 | 83 | 220 |
|  | Vz/F (L) | 485.0 | 471.0 | 120.9 | 24.9 | 25.0 | 468.1 | 292.9 | 723.9 |
|  | t½ (h) | 14.7 | 14.3 | 3.5 | 23.7 | 24.7 | 14.5 | 7.9 | 24.2 |
|  | T_LAG_ (h) | NA | NA | NA | NA | NA | 0.5 | 0.00 | 2.00 |
|  | T_MAX_ (h) | NA | NA | NA | NA | NA | 4.0 | 1.98 | 9.98 |
| TRV045 150 mg | AUC_INF_ (h*ng/mL) | 6840.5 | 6575.9 | 1959.0 | 28.6 | 30.0 | 6696.2 | 2667.8 | 13196.4 |
|  | AUC_LAST_ (h*ng/mL) | 6077.0 | 5908.7 | 1358.2 | 22.3 | 26.0 | 6027.9 | 2481.0 | 8800.0 |
|  | CL/F (L/h) | 23.9 | 22.8 | 8.3 | 34.7 | 30.0 | 22.4 | 11.4 | 56.2.0 |
|  | C_MAX_ (ng/mL) | 446.8 | 432.7 | 105.7 | 23.7 | 27.9 | 443.0 | 198.0 | 673 |
|  | Vz/F (L) | 492.5 | 472.2 | 164.4 | 33.4 | 28.9 | 491.6 | 297.5 | 1119.8 |
|  | t½ (h) | 15.0 | 14.4 | 5.1 | 34.3 | 28.5 | 13.8 | 9.2 | 35.5 |
|  | T_LAG_ (h) | NA | NA | NA | NA | NA | 0.50 | 0.00 | 1.00 |
|  | T_MAX_ (h) | NA | NA | NA | NA | NA | 5.98 | 1.98 | 10.02 |
| TRV045 300 mg | AUC_INF_ (h*ng/mL) | 12035.5 | 11457.0 | 3577.7 | 29.7 | 35.1 | 11829.0 | 3680.5 | 20035.9 |
|  | AUC_LAST_ (h*ng/mL) | 10679.7 | 10254.4 | 2812.8 | 26.3 | 32.0 | 10624.3 | 3454.4 | 17049.8 |
|  | CL/F (L/h) | 27.8 | 26.2 | 12.8 | 45.7 | 35.1 | 25.4 | 15.0 | 81.5 |
|  | C_MAX_ (ng/mL) | 682.9 | 655.1 | 182.2 | 26.7 | 32.2 | 680 | 227 | 1030 |
|  | Vz/F (L) | 571.0 | 539.1 | 235.2 | 41.2 | 33.2 | 513.6 | 334.2 | 1498.7 |
|  | t½ (h) | 14.7 | 14.3 | 4.1 | 28.1 | 25.4 | 14.0 | 9.3 | 29.4 |
|  | T_LAG_ (h) | NA | NA | NA | NA | NA | 0.5 | 0.0 | 1.0 |
|  | T_MAX_ (h) | NA | NA | NA | NA | NA | 6.0 | 4.0 | 10.0 |

AUC_INF_ is the area under the concentration‑time curve from time zero to infinity; AUC_LASTt_ is the area under the concentration-time curve from time zero to time of last quantifiable concentration; CL/F the apparent total clearance following extravascular administration; C_MAX_ the maximum plasma concentration, occurring at T_MAX_; CV the coefficient of variation; Max: maximum; Min: minimum; SD = standard deviation; t½ = terminal half‑life.

**Supplemental Table 2:** Results of evoked pain tests in all treatment groups

|  | **Estimated mean difference *versus* placebo (95% confidence interval)** | | |
| --- | --- | --- | --- |
| **Evoked pain tests** | **TRV045 50 mg (*n* = 25)** | **TRV045 150 mg (*n* = 25)** | **TRV045 300 mg (*n* = 25)** |
| Secondary mechanical allodynia:  surface areas (mm^2^) | -59.3 (-247.4 to 128.82)  p = 0.531 | -304.1 (-494.8 to -113.4)  p = 0.002 | -298.5 (-486.8 to -110.2)  p = 0.002 |
| Total mechanical allodynia:  surface area (mm^2^) | -200.92 (-500.0 to 98.1)  p = 0.184 | -595.1 (-902.0 to -288.6)  p = 0.0002 | -616.36 (-917.0 to -315.8)  p = 0.0001 |
| Electrical pain (stair paradigm):  pain detection threshold (mA) | -17.9% (-29.4% to -4.6%)  p = 0.011 | -12.7% (-24.7% to 1.2%)  p = 0.071 | -8.9% (-21.4% to 5.7%)  p = 0.215 |
| Electrical pain (stair paradigm):  pain tolerance threshold (mA) | -1.8% ( -9.2% to 6.1%)  p = 0.637 | -1.8% ( -9.0% to 5.9%)  p = 0.625 | 2.5% ( -5.0% to 10.6%)  p=0.513 |
| Electrical burst test:  pain detection threshold (mA) | -7.1% (-19.2% to 6.8%)  p = 0.295 | -3.1% (-15.7% to 11.4%)  p = 0.650 | 1.9% (-11.6% to 17.5%)  p = 0.7886 |
| Electrical burst test:  pain tolerance threshold (mA) | -1.5% ( -7.3% to 4.5%)  p = 0.606 | 3.1% ( -2.9% to 9.4%)  p = 0.314 | 2.7% ( -3.3% to 9.0%)  p = 0.379 |
| Cold pressor test:  pain detection threshold (s) | 8.1% (-10.9% to 31.2%)  p = 0.422 | 1.9% (-16.0% to 23.7%)  p = 0.844 | 10.8% ( -8.7% to 34.5%)  p = 0.295 |
| Cold pressor test:  pain tolerance threshold (s) | 0.7% ( -7.4% to 9.5%)  p = 0.875 | 5.5% ( -3.0% to 14.7%)  p =0.206 | 8.6% ( -0.1% to 18.1%)  p = 0.053 |
| Pressure pain test:  pain detection threshold (kPa) | -4.5% (-15.6% to 8.2%)  p = 0.463 | -6.0% (-17.0% to 6.5%)  p = 0.321 | 0.2% (-11.6% to 13.5%)  p = 0.979 |
| Pressure pain test:  pain tolerance threshold (kPa) | -4.3% (-11.0% to 2.9%)  p = 0.228 | -1.0% ( -7.9% to 6.6%)  p = 0.792 | 4.5% ( -2.9% to 12.5%)  p = 0.237 |
| Heat pain test - normal skin, upper back:  pain detection threshold (°C) | 0.08 ( -0.52 to 0.69)  p = 0.782 | 0.47 ( -0.14 to 1.07)  p = 0.127 | 0.24 ( -0.36 to 0.85)  p =0.425 |
| Heat pain - UVB skin, upper back):  pain detection threshold (°C) | 0.31 ( -0.20 to 0.82)  p = 0.231 | 0.05 ( -0.46 to 0.57)  p = 0.836 | 0.31 ( -0.20 to 0.83)  p = 0.229 |
| Heat pain test - capsaicin skin, volar forearm: pain detection threshold (°C) | 0.07 ( -0.46 to 0.60)  p = 0.788 | 0.35 ( -0.18 to 0.89)  p = 0.191 | 0.44 ( -0.08 to 0.97)  p = 0.099 |
| Heat pain test - normal skin, volar forearm: pain detection threshold (°C) | 0.02 ( -0.42 to 0.47)  p = 0.922 | 0.10 ( -0.35 to 0.55)  p = 0.651 | 0.37 ( -0.08 to 0.82)  p = 0.109 |

**Supplemental Table 3:** Adverse events by Medical Dictionary for Regulatory Activities (MedDRA) system organ class and preferred term (number (%)).

|  | Placebo  (n = 25) | | TRV045 50 mg  (n = 25) | | TRV045 150 mg  (n = 25) | | TRV045 300 mg  (n = 25) | |
| --- | --- | --- | --- | --- | --- | --- | --- | --- |
| System organ class | **Subjects** | **AE** | **Subjects** | **AE** | **Subjects** | **AE** | **Subjects** | **AE** |
| **Blood and lymphatic system disorders** | 1 (4%) | 1 | 0 | 0 | 2 (8%) | 2 | 0 | 0 |
| Anemia | 0 | 0 | 0 | 0 | 1 (4%) | 1 | 0 | 0 |
| Lymphadenopathy | 1 (4%) | 1 | 0 | 0 | 1 (4%) | 1 | 0 | 0 |
| **Cardiac disorders** | 1 (4%) | 1 | 0 | 0 | 0 | 0 | 0 | 0 |
| Palpitations | 1 (4%) | 1 | 0 | 0 | 0 | 0 | 0 | 0 |
| **Eye disorders** | 0 | 0 | 0 | 0 | 2 (8%) | 2 | 0 | 0 |
| Ocular discomfort | 0 | 0 | 0 | 0 | 1 (4%) | 1 | 0 | 0 |
| Vision blurred | 0 | 0 | 0 | 0 | 1 (4%) | 1 | 0 | 0 |
| **Gastrointestinal disorders** | 0 | 0 | 1 (4%) | 2 | 1 (4%) | 1 | 2 (8%) | 2 |
| Dry mouth | 0 | 0 | 0 | 0 | 0 | 0 | 1 (4%) | 1 |
| Nausea | 0 | 0 | 1 (4%) | 2 | 1 (4%) | 1 | 1 (4%) | 1 |
| **General disorders and administration site conditions** | 4 (16%) | 5 | 5 (20%) | 6 | 6 (24%) | 6 | 4 (16%) | 4 |
| Application site pruritus | 0 | 0 | 1 (%) | 1 | 0 | 0 | 0 | 0 |
| Application site wound | 0 | 0 | 1 (4.%) | 1 | 0 | 0 | 0 | 0 |
| Chills | 1 (4%) | 1 | 1 (4%) | 1 | 0 | 0 | 0 | 0 |
| Fatigue | 3 (12%) | 3 | 1 (4%) | 1 | 3 (12%) | 3 | 2 (8%) | 2 |
| Feeling abnormal | 0 | 0 | 0 | 0 | 1 (4%) | 1 | 0 | 0 |
| Feeling of relaxation | 0 | 0 | 0 | 0 | 1 (4%) | 1 | 1 (4%) | 1 |
| Medical device site bruise | 0 | 0 | 0 | 0 | 1 (4%) | 1 | 0 | 0 |
| Medical device site erythema | 0 | 0 | 1 (4%) | 1 | 0 | 0 | 0 | 0 |
| Medical device site injury | 0 | 0 | 0 | 0 | 0 | 0 | 1 (4%) | 1 |
| Medical device site irritation | 0 | 0 | 1 (4%) | 1 | 0 | 0 | 0 | 0 |
| Vessel puncture site hematoma | 1 (4%) | 1 | 0 | 0 | 0 | 0 | 0 | 0 |
| **Infections and infestations** | 0 | 0 | 1 (4%) | 1 | 0 | 0 | 1 (4%) | 1 |
| Gastroenteritis | 0 | 0 | 1 (4%) | 1 | 0 | 0 | 0 | 0 |
| Viral infection | 0 | 0 | 0 | 0 | 0 | 0 | 1 (4%) | 1 |
| **Injury, poisoning and procedural complications** | 0 | 0 | 0 | 0 | 1 (4%) | 1 | 0 | 0 |
| Procedural pain | 0 | 0 | 0 | 0 | 1 (4%) | 1 | 0 | 0 |
| **Musculoskeletal and connective tissue disorders** | 0 | 0 | 0 | 0 | 1 (4%) | 1 | 0 | 0 |
| Back pain | 0 | 0 | 0 | 0 | 1 (4%) | 1 | 0 | 0 |
| **Nervous system disorders** | 9 (36%) | 10 | 5 (20%) | 6 | 12 (48%) | 21 | 15 (60%) | 22 |
| Allodynia | 0 | 0 | 0 | 0 | 1 (4%) | 1 | 0 | 0 |
| Amnesia | 1 (4%) | 1 | 0 | 0 | 0 | 0 | 0 | 0 |
| Balance disorder | 0 | 0 | 0 | 0 | 0 | 0 | 1 (4%) | 1 |
| Disturbance in attention | 1 (4%) | 1 | 0 | 0 | 2 (8%) | 2 | 0 | 0 |
| Dizziness | 2 (8%) | 2 | 0 | 0 | 3 (12%) | 3 | 3 (12%) | 3 |
| Headache | 2 (8%) | 2 | 3 (12%) | 3 | 8 (32%) | 9 | 8 (32%) | 9 |
| Paraesthesia | 1 (4%) | 1 | 0 | 0 | 0 | 0 | 1 (4%) | 1 |
| Presyncope | 0 | 0 | 1 (4%) | 1 | 0 | 0 | 0 | 0 |
| Sedation | 0 | 0 | 0 | 0 | 1 (4%) | 1 | 0 | 0 |
| Somnolence | 2 (%) | 3 | 2 (8%) | 2 | 5 (20%) | 5 | 8 (32%) | 8 |
| **Psychiatric disorders** | 1 (4.%) | 1 | 0 | 0 | 1 (4%) | 1 | 1 (4%) | 1 |
| Abnormal dreams | 1 (%) | 1 | 0 | 0 | 0 | 0 | 0 | 0 |
| Agitation | 0 | 0 | 0 | 0 | 1 (4%) | 1 | 0 | 0 |
| Bradyphrenia | 0 | 0 | 0 | 0 | 0 | 0 | 1 (4%) | 1 |
| **Respiratory, thoracic and mediastinal disorders** | 0 | 0 | 0 | 0 | 3 (12%) | 3 | 0 | 0 |
| Cough | 0 | 0 | 0 | 0 | 1 (4%) | 1 | 0 | 0 |
| Epistaxis | 0 | 0 | 0 | 0 | 1 (4%) | 1 | 0 | 0 |
| Oropharyngeal pain | 0 | 0 | 0 | 0 | 1 (4%) | 1 | 0 | 0 |
| **Skin and subcutaneous tissue disorders** | 0 | 0 | 1 (4%) | 1 | 0 | 0 | 0 | 0 |
| Hyperhidrosis | 0 | 0 | 1 (4%) | 1 | 0 | 0 | 0 | 0 |
| **Vascular disorders** | 1 (4%) | 1 | 1 (4%) | 1 | 0 | 0 | 0 | 0 |
| Orthostatic hypotension | 1 (4%) | 1 | 1 (4%) | 1 | 0 | 0 | 0 | 0 |

Data are number of subjects (%), number of adverse events (AE)
